# Listeriosis outbreak caused by contaminated stuffed pork, Andalusia, Spain, July to October 2019

**DOI:** 10.2807/1560-7917.ES.2022.27.43.2200279

**Published:** 2022-10-27

**Authors:** Nicolás Francisco Fernández-Martínez, Rafael Ruiz-Montero, Eduardo Briones, Elena Baños, Lucía García San Miguel Rodríguez-Alarcón, J. Alberto Chaves, Raquel Abad, Carmen Varela, on behalf of the LISMOAN team, Nicola Lorusso

**Affiliations:** 1 Preventive Medicine and Public Health Unit, Reina Sofia University Hospital, Córdoba, Spain; 2 Preventive Medicine and Public Health Research Group, Maimonides Biomedical Research Institute of Cordoba (IMIBIC), Córdoba, Spain; 3 Department of Medical and Surgical Sciences, University of Córdoba, Córdoba, Spain; 4 Public Health Unit, Sevilla Health District, Sevilla, Spain; 5 CIBER in Epidemiology and Public Health (CIBERESP), Sevilla, Spain; 6 Directorate General of Public Health and Pharmaceutical Management, Regional Ministry of Health and Consumer Affairs, Sevilla, Spain; 7 Coordinating Centre for Health Alerts and Emergencies (CCAES), Directorate General of Public Health, Ministry of Health, Madrid, Spain; 8 Neisseria, Listeria and Bordetella Unit, National Centre for Microbiology, Instituto de Salud Carlos III, Majadahonda, Madrid, Spain; 9 National Centre of Epidemiology, Carlos III Health Institute, Madrid, Spain; 10 CIBER of Epidemiology and Public Health (CIBERESP), Madrid, Spain; 11 Members of the LISMOAN team are listed under Acknowledgements

**Keywords:** Listeria monocytogenes, disease outbreaks, foodborne diseases, listeriosis, communicable disease control, food contamination, molecular epidemiology

## Abstract

Between 1 July and 26 October 2019 in Andalusia, Spain, a large outbreak with 207 confirmed cases of listeriosis was identified. Confirmed cases had a median age of 44 years (range: 0–94) and 114 were women (55.1%). Most cases (n = 154) had mild gastroenteritis, 141 (68.1%) required hospitalisation and three died; five of 34 pregnant women had a miscarriage. The median incubation period was 1 day (range: 0–30), and was significantly shorter in cases presenting with gastroenteritis compared to those presenting without gastroenteritis (1 day vs. 3 days, respectively, p value < 0.001). Stuffed pork, a ready-to-eat product consumed unheated, from a single producer contaminated with *Listeria monocytogenes* ST388 was identified as the source of infection. The outbreak strain was identified in 189 human samples and 87 non-human (82 food and 5 environmental) samples. Notification of new cases declined abruptly after control measures were implemented. These included contaminated food recall, protocols for clinical management of suspected cases and for post-exposure prophylaxis in pregnant women and communication campaigns with concise messages to the population through social media. Given that there were 3,059 probable cases, this was the largest *L. monocytogenes* outbreak ever reported in Europe.

**Key public health message**

**What did you want to address in this study?**

*Listeria* *monocytogenes* is a pathogen responsible for food-borne outbreaks, particularly dangerous for pregnant women and immunocompromised individuals. We wished to explore the epidemiological characteristics, causative agent and source of infection of a large outbreak caused by *L. monocytogenes* in a region of southern Spain.

**What have we learnt from this study?**

A total of 207 microbiologically confirmed cases and over 3,000 suspected cases were detected during the outbreak from July to October 2019, which was linked to contaminated stuffed pork. We observed a considerable impact on pregnant women and neonates.

**What are the implications of your findings for public health?**

Stuffed pork should be considered a new risk food. It is important to reinforce food safety, especially in a society where an increasing amount of ready-to-eat food is consumed. In addition, field epidemiology is of great value for the early detection of outbreaks.

## Background

*Listeria monocytogenes* is a ubiquitous, facultative intracellular, Gram-positive bacillus and the pathogen responsible for the disease listeriosis. Consumption of contaminated food is the most important transmission route in listeriosis outbreaks. *L. monocytogenes* can persist under adverse environmental conditions, including low temperatures (i.e. refrigerators) and multiply in various foods, such as pasteurised and unpasteurised dairy products (milk, soft cheese), meat products (sausages, pâté), fresh fruits and vegetables (lettuce), and fish products (smoked salmon) [1]. 

The clinical spectrum of listeriosis ranges from asymptomatic infection and febrile gastroenteritis, often self-limited, to invasive disease, with sepsis and meningoencephalitis as the main clinical syndromes. Risk factors include advanced age, pregnancy and situations that weaken the immune system, such as cancer, transplantation, untreated HIV infection, steroid therapy and chronic diseases [2]. Less frequent than food-borne cases– but with great clinical impact – are cases of vertical transmission, which occur primarily when the pathogen crosses the placental barrier [3]. Pregnancy-associated listeriosis deserves special attention as foetal infection is frequent and chorioamnionitis can result in neonatal meningitis, characterised by high mortality rates despite appropriate treatment.

The incidence of listeriosis has been steadily increasing in Europe since 2008 (with the exception of 2020, likely on account of under-notification during the COVID-19 pandemic). In 2020, Estonia and Iceland were the European Union/European Economic Area (EU/EEA) countries with the highest age-standardised rate (1.39 and 1.19 per 100,000 population, respectively) [4]. Based on hospitalisation records, Spain has experienced a rising trend since 1997. For example, during the period 1997–2015, the hospitalisation rate in the age group ≥ 65 years increased from 0.5 per 100,000 population to almost 3.0 per 100,000 [5]. In Spain, notification of listeriosis cases is mandatory. In the study region Andalusia, an autonomous community in southern Spain, listeriosis has been a notifiable disease since 1996, since development of its epidemiologic surveillance system (SVEA, Epidemiological Surveillance System of Andalusia), which is integrated within the national surveillance network.

## Outbreak detection

On 5 August 2019, local primary care epidemiologists at the Aljarafe Health District in the province of Seville, Andalusia, notified to the SVEA three cases of food poisoning that had occurred in the previous week. One case was hospitalised; this patient’s blood culture later revealed *L. monocytogenes* growth. In the following days, five additional clusters comprising a total of 32 cases were notified in Seville and one adjacent Andalusian province, and the same pathogen was identified in two of those clusters. These events prompted the local health authorities’ response on 7 August, with instructions to intensify the epidemiological surveillance and environmental sampling in certain food establishments.

By 9 August, the aggregate patient count had reached 22, which included four other cases requiring hospitalisation. In the following days, given the information from epidemiological interviews, in conjunction with microbiological results from non-human samples, the regional government constituted a multidisciplinary committee to investigate the outbreak, comprised of professionals from epidemiological surveillance, healthcare (including urgent care and infectious diseases), microbiology, food safety, and health authorities. 

The aim of the outbreak investigation was twofold: (i) to identify the causative agent and the source of infection in order to prevent any further cases and (ii) to discover the reasons behind such a rapid progression of the outbreak. Here, we describe the outbreak and the epidemiological, microbiological and environmental investigations that were carried out to answer these questions.

## Methods

### Case definitions

The outbreak time window ranged from 1 July to 26 October 2019. The start of the outbreak was defined by a sustained increase in new weekly cases (more than three weekly cases for at least 2 consecutive weeks) and the end was defined by 70 days (the maximum incubation period for listeriosis) after contaminated food products were recalled. 

Confirmed cases were defined as patients who, within this period, had a sample in which of *L. monocytogenes* was detected with genomic confirmation by whole genome sequencing (WGS) or symptoms compatible with listeriosis and history of consumption of potentially contaminated products (any meat product from Facility X or stuffed pork from any facility). Probable cases were defined as patients who, within this period, showed clinical-epidemiological criteria, but not detection of *L. monocytogenes*. Criteria for case definitions are summarised in Table 1.

**Table 1:** Clinical, microbiological and epidemiological case definition criteria for the listeriosis outbreak, Andalusia, Spain, 1 July–26 October 2019

Case definition criteria		Type of case associated		
		Confirmed (by WGS)	Confirmed	Probable
Clinical	Criterion A: any symptom compatible with listeriosis^a^	Not required	X	X
Microbiological	Criterion A: isolation of *L. monocytogenes* in a normally sterile site (or faeces, if high clinical epidemiological suspicion)	X	X	NA
	Criterion B1: genome related to the outbreak strain (CC388; ST-388; CT-8466)	X	NA	NA
	Criterion B2: genome not available	NA	X	X
Epidemiological	Criterion A: consumption of any meat product from Facility X or stuffed pork from any facility in the 70 days previous to the start of symptoms	Not required	X	X
	Criterion B: start of symptoms or date of diagnosis compatible with the outbreak period (1 July–26 October 2019)	X	X	X

CC: clonal complexes; CT: cluster type; *L.*: *Listeria*; NA: not applicable; ST: sequence type; WGS: whole genome sequencing; X: criterion required to meet a specific case definition.

^a^ Symptoms compatible with listeriosis include: (i) in all cases, acute gastroenteritis, fever, influenza-like illness, meningitis or meningoencephalitis, sepsis and localised infections (such as abscesses, arthritis and endocarditis); (ii) in pregnant women, spontaneous abortion, stillbirth and premature birth; and (iii) in newborn infants, granulomatosis infantiseptica, meningitis or meningoencephalitis, sepsis, dyspnoea, and mucocutaneous lesions.

Invasive listeriosis was defined as listeriosis presenting with isolation of *L. monocytogenes* from a normally sterile site or, in the setting of pregnancy-associated cases, from placental or foetal tissue.

Gestational cases included listeriosis onset during pregnancy causing any of the following: (i) pregnancy loss (encompassing spontaneous abortion, before the 20th gestational week and stillbirth, after this period), (ii) premature birth (before the 37th week) or (iii) newborn *L. monocytogenes* infection.

Neonatal cases included illness with an onset within the first 30 days of life causing at least one of the following: (i) granulomatosis infantiseptica, (ii) meningitis or meningoencephalitis, (iii) sepsis, (iv) dyspnoea and (v) mucocutaneous lesions. Association of neonatal cases to this outbreak was assessed individually, irrespective of the date of onset of symptoms (hereafter, illness onset).

Immunosuppression was defined as solid organ transplantation, haematopoietic stem-cell transplantation, solid cancer with metastases, solid cancer undergoing chemotherapy and/or radiotherapy, haematologic malignancies, severe neutropenia (< 500 neutrophils/µL), stage IV chronic kidney disease, primary immunodeficiencies (except IgA deficit), HIV infection with lymphocyte CD4+ T-cell count below 200 cells/ml in the 6 months prior to hospitalisation, and chronic inflammatory diseases treated with high-level immunosuppressive therapy [6].

Sequelae were defined as persistent signs or symptoms present at least 3 months after hospital discharge or, in the event of gestational listeriosis, pregnancy loss.

### Epidemiological investigation

#### Questionnaire

The clinical–epidemiological information on cases (i.e. symptoms, food consumption, incubation period, hospitalisation) was taken from two sources: the SVEA registries, which collect case-based epidemiological data, and digital medical records from the Andalusian Health Service, from which complementary clinical details were abstracted with the use of a standardised case investigation form. Epidemiologists from the SVEA attempted to interview all patients with confirmed or probable listeriosis within 24 h, using a standardised questionnaire (Supplement S1 contains the SVEA epidemiological questionnaire.). The questionnaire included epidemiological, clinical and laboratory information, as well as a series of questions regarding food exposures (meat products, milk and dairies, fish and seafood, vegetables, pre-cooked dishes) in the 21 days before illness onset. Both the patient’s diet and food eaten at special events were assessed. If the patient was a child (aged ≤ 12 years) or was unable to respond, the next of kin was interviewed. Certain sections, such as food exposures and case definition criteria, were modified over the course of the outbreak in August 2019, to adapt the questionnaire to the outbreak investigation (Supplement S1: SVEA epidemiological questionnaire).

#### Active case finding

Prospective and retrospective active case finding was conducted. Health authorities advised the general population to avoid consumption of foods that were suspected as a possible vehicle (see below) and encouraged individuals who developed symptoms of listeriosis to seek medical care. Outbreak updates were shared with SVEA epidemiologists and healthcare workers to improve detection of new cases; all available *L. monocytogenes* isolates from 2017 to 2019 were sequenced by WGS.

Information about possible outbreak-related cases was also collected from other regions in Spain as well as other countries from the national surveillance database (SiViEs) and communication platform with EU/EEA (Early Warning and Response System (EWRS)). At the national level, any case with a sample, including stool, with the outbreak strain (IVb; CC388; ST-388; CT-8466) was considered as a confirmed outbreak case (this definition differed slightly from that of the study region). The cases detected at the national and international levels are briefly described in a separate section.

#### Statistical analysis

To compare the length of the incubation period between groups with different clinical presentations, the Wilcoxon rank sum test was used. To analyse factors associated with the length of the incubation period, a multivariable logistic regression model was applied, adjusting for sex, age and days elapsed since the outbreak start. We hypothesised that a larger number of days elapsed since the outbreak start might entail a higher degree of contamination and thus a shorter incubation period, whereas the opposite would be true for some clinical forms (e.g. gestational listeriosis). Collinearity of the model was measured by the variance inflation factors, setting the threshold of non-collinearity at < 2.5. Calibration of the model was assessed by the Hosmer-Lemeshow test. Significance of the regression coefficients was assessed by the Wald’s test, with p values ≥ 0.05 considered significant. The analyses were performed using R software version 4.1.0 [7].

### Microbiological investigation

#### Clinical sample collection and whole genome sequencing 

Human samples (e.g. blood, cerebrospinal fluid, placenta, stool specimens) are collected upon clinical suspicion of listeriosis by healthcare providers in Andalusia. For this outbreak investigation, all available *L. monocytogenes* isolates from confirmed cases between 2017 and 2019 in Andalusia were forwarded to the National Centre of Microbiology (CNM) for sequencing purposes. Briefly, the process consisted of extracting DNA from a pure culture, which was then quantified and qualified, preparing paired-end libraries and performing WGS on the MiSeq or NextSeq platforms (Illumina). Once the genome was sequenced, the reads were de novo assembled to characterise the isolates. Characterisation entailed: (i) identification of the serogroup/serotype via multiplex in silico PCR assay [8,9], (ii) sequence type (ST) determination via multilocus sequence typing (MLST), combining fragments of seven genes [10], (iii) clonal complex identification [10], (iv) cluster type (CT) determination, following Ruppitsch’s core genome MLST scheme, which contains an allelic profile of 1701 genes [11] and (v) genome comparison, defining as potentially related isolates those with four or fewer differences in the allelic profile, according to core genome MLST [12]. Not all human samples were subject to laboratory analyses as a minority of samples were contaminated or did not have sufficient material to perform WGS.

#### Food and environmental trace-back investigations

Food and environmental samples (from potentially exposed surfaces) were obtained from food establishments, including factories, markets and grocery stores, bars and restaurants; some samples were provided by consumers from their homes, when available. These investigations were guided by the places where the suspected food products were produced, sold or consumed. An inspection was conducted in the main facility suspected as the source of infection, in which sampling included raw materials, processed foods, work surfaces, food processing equipment and other types of machinery. Traceback investigations were based on bacteria detection according to the ISO 11290–2 method, which specifies that quantification of colony-forming units per gram (CFU/g) is mandatory in ready-to-eat (RTE) food products that do not support the growth of *L. monocytogenes*; and/or the ISO 11290–1 method, which consists of determining the absence or presence of *L. monocytogenes*. Altogether, more than 200 non-human samples were taken during the outbreak investigation. To confirm the association between confirmed cases and the suspected food vehicle, CNM also performed WGS in certain food samples following the process described above. 

## Results

### Descriptive epidemiology

**Figure 1:**
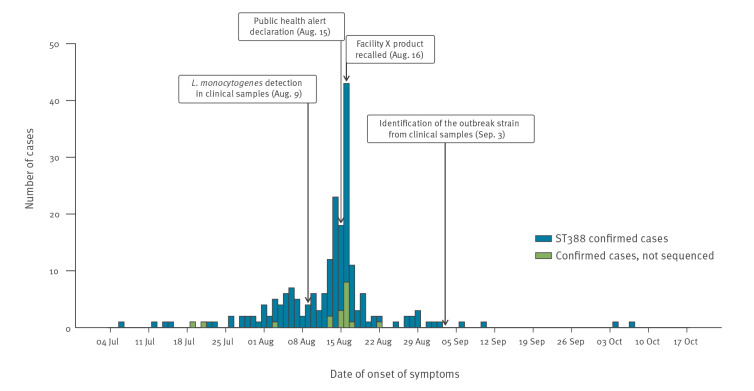
Confirmed cases of listeriosis by date of symptom onset, Andalusia, Spain, 1 July–26 October 2019 (n = 207)

**Figure 2:**
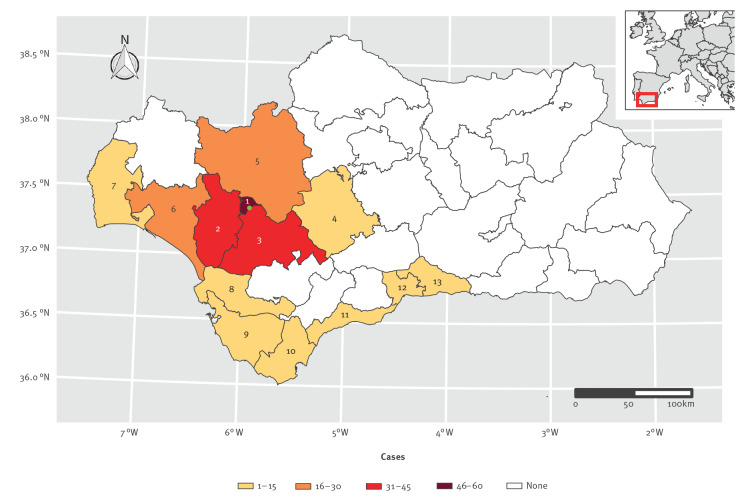
Spatial distribution of confirmed cases of listeriosis, Andalusia, Spain, 1 July–26 October 2019 (n = 207)

During the outbreak period in Andalusia, 3,582 suspected cases were investigated: 207 were confirmed and 3,059 were probable; 316 cases were excluded because they did not fulfil the case definition criteria. Of the confirmed cases, 197 (95.2%) were interviewed, most within 48 h of being notified. Symptom onset dates ranged from 7 July to 7 October 2019, reaching the peak on 16 August with 43 cases (Figure 1). Confirmed cases had a median age of 44 years (range: 0–94) and 114 were women (55.1%). Their demographic and epidemiological characteristics are shown in Table 2. Despite four provinces being affected, most confirmed cases occurred in Seville (78.7%) (Figure 2).

Facility X: production site suspected to be the source of the contaminated food products responsible for the listeriosis outbreak.

**Table 2:** Epidemiological characteristics of confirmed cases of listeriosis, Andalusia, Spain, 1 July–26 October 2019 (n = 207)

Variable	Confirmed cases					
	Total		Female		Male	
	n	%	n	%	n	%
Number of cases	**207**	**100**	**114**	**55.1**	**93**	**44.9**
Age, years						
median (IQR)	44	33–61	38	31–58	47	35–64
0–4	8	3.9	0	0	8	8.6
5–19	11	5.3	9	7.9	2	2.2
20–29	25	12.1	17	14.9	8	8.6
30–39	45	21.7	32	28.1	13	14.0
40–49	36	17.4	17	14.9	19	20.4
50–59	26	12.6	12	10.5	14	15.1
60–69	24	11.6	12	10.5	12	12.9
70–79	19	9.2	8	7.0	11	11.8
80–100	13	6.3	7	6.1	6	6.5
Province						
Seville	163	78.7	95	83.3	68	73.1
Huelva	29	14.0	13	11.4	16	17.2
Cadiz	11	5.3	2	1.8	9	9.7
Malaga	4	1.9	4	3.5	0	0
Food exposure^a^						
Stuffed pork	186	89.9	105	92.1	81	87.1
Chicken/turkey	1	0.5	1	0.9	0	0
Cured ham	1	0.5	1	0.9	0	0
Doner kebab	1	0.5	0	0	1	1.1
Pepper beef	1	0.5	1	0.9	0	0
Boiled ham with cheese	1	0.5	0	0	1	1.1
Unknown	16	7.7	6	5.3	10	10.8
Producer^a^						
Facility X	127	61.4	75	65.8	52	55.9
Another producer	1	0.5	1	0.9	0	0
Unknown	79	38.2	38	33.3	41	44.1

IQR: interquartile range.

^a^ Missing values are presented as ‘unknown’.

Health districts are marked as follows: 1. Seville, 2. Aljarafe, 3. South Seville, 4. East Seville, 5. North Seville, 6. Condado-Campiña, 7. Huelva-Costa, 8. Jerez-Costa Noroeste, 9. Bahía de Cádiz-La Janda, 10. Campo de Gibraltar, 11. Costa del Sol, 12. Malaga, 13. Axarquia. Health districts 1 to 5 belong to the province of Seville, 6 and 7 belong to the province of Huelva, 8 to 10 belong to the province of Cadiz and 11 to 13 belong to the province of Malaga. The location of Facility X is indicated by a green dot. See Supplement S4 for the incidence per 100,000 population, by health district.

**Figure 3:**
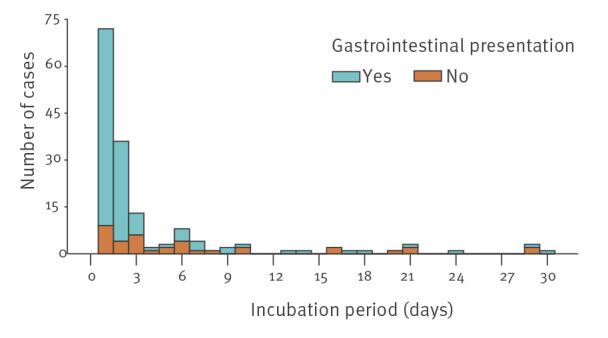
Duration of the incubation period among confirmed cases of listeriosis with (n = 154) and without (n = 53) gastrointestinal presentation, Andalusia, Spain, 1 July–26 October 2019

The incubation period was assessed in all confirmed cases for whom information about the date of illness onset and the date of food exposure were available (n = 179, 86.5%), yielding a median of 1 day (interquartile range (IQR): 1–3 days, range: 0–30). There were statistically significant differences (p value of Wilcoxon rank sum test < 0.001) between confirmed cases of listeriosis presenting with gastroenteritis (n = 154, median: 1 day, IQR: 1–2) and without gastroenteritis (n = 53, median: 3 days, IQR: 1–7.5). Figure 3 represents the distribution of the incubation period among both subgroups. The variables ‘gastrointestinal presentation’ and ‘a higher number of days elapsed since the outbreak started’ were independently associated with a shorter incubation period (of 2 days or fewer) in a multivariable logistic regression model, adjusted by age, sex, and immunosuppression (Supplement S2 shows the multivariable logistic regression model).

### Clinical features

An invasive clinical form was diagnosed in almost all patients with confirmed listeriosis (n = 194, 93.7%) and two in three required hospitalisation (n = 141, 68.1%). Gestational listeriosis was observed in 14 patients (6.8%), similar to neonatal listeriosis (n = 11, 5.3%). Immunosuppression (10.1%), diabetes mellitus (9.7%) and cancer (4.3%) constituted the main underlying conditions. The outcome was known in 203 patients (98.1%). Among these, most were cured (91.6%), while 6.9% developed sequelae, and three died (overall case-fatality rate (CFR): 1.5%). Specifically, of the 34 pregnant women, five experienced a pregnancy loss. Clinical features are shown in Table 3.

**Table 3:** Clinical features of confirmed cases of listeriosis, Andalusia, Spain, 1 July–26 October 2019 (n = 207)

Variables	Confirmed cases					
	Total n = 207		Female n = 114		Male n = 93	
	n	%	n	%	n	%
Predisposing factors						
Pregnancy	34	16.4	34	29.8	NA	NA
Immunosuppression	21	10.1	12	10.5	9	9.7
Diabetes mellitus	20	9.7	10	8.8	10	10.8
Cancer	9	4.3	3	2.6	6	6.5
Liver cirrhosis	2	1.0	0	0	2	2.2
HIV infection^a^	1	0.5	0	0	1	1.1
Clinical form						
Invasive listeriosis	194	93.7	110	96.5	84	90.3
- Gestational listeriosis	14	6.8	14	12.3	NA	NA
- Neonatal listeriosis	11	5.3	5	4.4	6	6.5
- Meningoencephalitis or sepsis	28	13.5	7	6.1	21	22.6
Gastrointestinal illness	154	74.4	91	79.8	63	67.7
Hospitalisation						
Number	141	68.1	87	76.3	54	58.1
Length of stay (median days, IQR)	9	6–15	8	6–14	12	6–21
Outcome^b^						
Cure	186	89.9	104	91.2	82	88.2
Sequelae^c^	14	6.8	7	6.1	7	7.5
Death^d^	3	1.4	2	1.8	1	1.1
Unknown	4	1.9	1	0.9	3	3.2

HIV: human immunodeficiency virus; IQR: interquartile range.

^a^ The case had a CD4^+^ T-cell count ˃ 200 cells/µL and therefore was not considered immunosuppressed.

^b^ Missing values are presented as ‘unknown’.

^c^ Sequelae, defined as persistent signs or symptoms present at least 3 months after hospital discharge or, in the event of gestational listeriosis, pregnancy loss, included: persistent hydrocephalus, persistent dysphagia, persistent arthralgias, muscle weakness, deterioration of general condition, refractory cytomegalovirus infection, stillbirth and spontaneous abortion.

^d^ All deaths recorded were reported in patients aged above 70 years.

### *Listeria monocytogenes* detection in human samples 

Clinical samples, i.e. biological matrix/tissues, taken during the outbreak in which *L. monocytogenes* was isolated were known in 173 patients (83.6%). Blood was, by far, the most frequent specimen collected from individuals (n = 145, 83.8%), followed by cerebrospinal fluid (n = 13, 7.5%), stool specimens (n = 5, 2.9%) and placental tissue (n = 4, 2.3%). Among asymptomatic patients (n = 15), *L. monocytogenes* was also primarily detected in blood (n = 12, 80%). However, in 34 samples (17.4%), the type of clinical sample was not known.

Through WGS of clinical samples taken upon clinical suspicion of listeriosis before the outbreak (between 2017 and 2019), active case finding identified 16 additional WGS-confirmed cases that preceded the outbreak start by several months (illness onset dates ranged from November 2018 to June 2019). Epidemiological interviews revealed that only one of these cases had consumed the contaminated meat product. Thus, they were deemed as historically associated cases and not included in the outbreak investigation; Supplement S3 shows the details of the epidemiological and clinical characteristics of historically associated cases.

### Epidemiological findings and investigation of infection source

**Figure 4:**
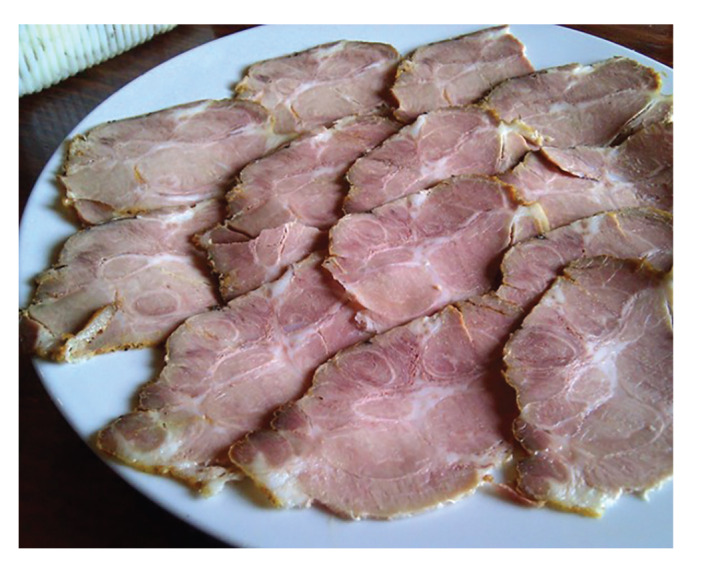
Representative image of stuffed pork similar to the type implicated in the listeriosis outbreak, Andalusia, Spain, 1 July–26 October 2019

Within Andalusia, most confirmed cases were distributed across the Seville health district and its neighbouring districts; Condado-Campiña in Huelva province accounted for the highest incidence of listeriosis with 15.6 cases per 100,000 population (Supplement S4 depicts the incidence of listeriosis by health district.). Food exposures in the 21 days before illness onset reported by cases from these districts led to an initial suspicion. Notably, 186 cases (94.4% of those who were interviewed) reported that they had eaten stuffed pork (a ready-to-eat food; a representative image shown in Figure 4), and 127 (64.5% of those interviewed) that they had eaten such food from the same brand (Table 2). Therefore, a specific item about stuffed pork consumption was added to the standardised questionnaire. When asked, most cases reported having purchased the stuffed pork at a variety of food establishments — primarily supermarkets and bars. Indeed, as one of the first confirmed cases occurred after the acquisition of stuffed pork at a grocery store, the trace-back investigation in that establishment pointed to a specific facility where it was produced, as described in the next section. Consumption of other foods was also reported, although to a lesser extent, with five cases reporting consumption of either chicken or turkey, cured ham, doner kebab, pepper beef or boiled ham with cheese, as shown in Table 2.

Stuffed pork is one of the most popular ‘tapas’ in the provinces affected by the outbreak. It consists of a piece of pork loin stuffed with garlic, a variety of spices and salt that is roasted for about 2 h until it is tender. Consumers usually keep stuffed pork in the refrigerator between 0^◦^C and 4^◦^C, as it is eaten as a cold cut (without previous heating).

Source: Attribution-non commercial-share alike 2.0 generic (CC BY-NC-SA 2.0). No changes were made to the image. Available from: https://www.flickr.com/photos/annalibera/6384563207/in/photostream

### Microbiological investigation

Serogroup identification and WGS data were available for 189 human samples (91.3%). All belonged to serogroup IVb and the outbreak strain was characterised as sequence type (ST) 388, clonal complex (CC) 388, and cluster type (CT) 8466.

### Environmental investigation

On 8 August, before detection of *L. monocytogenes* in clinical samples, a family reported gastrointestinal symptoms in some of its members after buying and consuming stuffed pork at a grocery store. The inspection of said store revealed that it sold stuffed pork from two brands, each produced by a different company, so both products of these two brands were sampled and tested for *L. monocytogenes*. One of the two samples tested positive for *L. monocytogenes*; we refer to the site where this sample was obtained as Facility X. There was an inspection at Facility X on 15 August 2019, but there were no contaminated samples in the initial Facility X inspection. However, there were nine contaminated non-human samples (four food and five environmental samples) from Facility X identified within the first 10 days. During the following 2 months, 55 additional food samples from Facility X were found to be contaminated. The exact dates of sampling in Facility X were not known, but most samples were taken between 15–30 August 2019.

*L. monocytogenes* colony-forming units per gram (CFU/g) were quantified in 42 food samples, finding values between 987 and 52x10^6^ CFU/g, well above the safety limit set by the European Union (EU) regulations (at 100 CFU/g [13]) and thus reflecting a high degree of contamination (Supplement S5 shows the CFU/g values of *L. monocytogenes* in the different contaminated food samples). Some tested samples were taken at Facility X, while the remainder were directly provided by consumers from their homes, where conditions for proper food preservation may not have been met.

### Coordination at the national and international levels

On 16 August 2019, the alert was notified to the Coordination Centre for Health Alerts and Emergencies (Spanish: CCAES), a body of the Spanish Ministry of Health in charge of the national coordination of public health alerts and national focal point of communication with the European Centre for Disease Prevention and Control (ECDC) and the World Health Organization (WHO). CCAES, together with CNM, National Centre of Epidemiology (CNE) and all autonomous communities, established a common protocol for the outbreak investigation and a case definition at the national level. An additional 10 outbreak cases from six different regions within Spain were communicated to SiViEs. Through the EWRS platform, French health authorities notified a confirmed case in an individual who had consumed pork meat in Seville on 13 August. In addition, on 5 August 2019, the National Focal Point from Spain contacted the National Focal Point of Germany through EWRS in order to verify the information about several cases of listeriosis communicated by the media. However, the German public health authorities could not verify such cases. 

## Outbreak control measures

Regional health authorities declared a public health alert on 15 August 2019. On the next day, cleaning and disinfection of Facility X were encouraged and an order was given to recall stuffed pork from Facility X, after which an abrupt reduction in the number of new confirmed cases of listeriosis was observed. On 19 August, production of any food was stopped in Facility X until safety could be ensured. On 20 August, the recall order was extended to any meat or meat derivative produced in Facility X, and three days later, the alert was updated to include every food product from Facility X. Parallel to food safety measures, two clinical management protocols were elaborated and disseminated among healthcare workers. The first protocol was for management of patients with suspected listeriosis. The second protocol was for post-exposure prophylaxis (PEP) with amoxicillin (after antimicrobial susceptibility testing excluded ampicillin-resistance in the outbreak strain) in pregnant women with a history of stuffed pork consumption, including a follow-up to evaluate whether they developed symptoms of listeriosis. During the period between 5–27 September 2019, more than 30,000 pregnant women were interviewed in Andalusia during the outbreak period and, upon individual assessment, ca 5% were recommended PEP. This protocol was developed for this specific outbreak in an attempt to limit the potential impact on pregnant women. 

The SVEA adapted its protocol for cases of listeriosis to the needs of the outbreak response. Alongside the public health alert, a communication campaign was launched in the national media on 15 August 2019 to raise public awareness of the outbreak. Clear and concise messages were disseminated through traditional and social media on a daily basis concerning the products affected by the alert, basic hygiene measures related to food preparation and consumption, symptoms compatible with listeriosis, guidance to pregnant women with a history of consumption of potentially contaminated products and general recommendations on when to seek medical care.

By the end of the outbreak, over 1,800 food establishments had been inspected, with *L. monocytogenes* contamination in 87 non-human samples. Most positive samples came from food products involving stuffed pork (n = 76) and other pork derivatives (n = 6). The remaining samples (n = 5) were environmental, mostly from machinery surfaces in Facility X. These included an oven trolley, meat injectors (metal syringes used to add condiments to meat) and an air conditioner vent. Regarding WGS, eight food samples were shown to harbour the outbreak strain.

The last confirmed case of listeriosis was reported on 7 October 2019. On 26 October, after 70 days had passed since the recall of potentially contaminated stuffed pork, the outbreak was declared over. The distribution of Facility X products was almost exclusively limited to the study region, yet the food recall was estimated to affect more than 8 tons of meat products. Of note, parallel to this outbreak, there were two other food alerts due to *L. monocytogenes* in the study region. WGS demonstrated that there were at least five allelic differences in the gene profiles and thus these alerts were unrelated to the outbreak (data not shown), but still posed difficulties for its control.

## Discussion

To our knowledge, we report the largest listeriosis outbreak in Spain and one of the largest in Europe to date. From 2018 to 2019, a large outbreak occurred in Germany, involving 112 confirmed cases of invasive listeriosis associated with contaminated blood sausage [14]. No European outbreak with 200 or more confirmed cases had been reported since 1992, when 279 confirmed cases of listeriosis related to jellied pork tongue consumption were recorded in France [15]. In the present study, contamination of stuffed pork from a single producer resulted in a large nationwide outbreak in Spain, affecting mainly four provinces in the Andalusia region over a period of 17 weeks. Once the outbreak was detected in early August, the food source was rapidly identified within 10 days through epidemiological interviews and later confirmed by WGS, which detected the outbreak strain (IVb; CC388; ST-388; CT-8466, available in the Listeria PasteurMLST database) in clinical isolates and non-human isolates from food samples taken at Facility X. Notification of new cases ceased after the implementation of early control measures, i.e. product recall, suggesting that food products from other facilities did not play a significant role in the outbreak development.

Invasive listeriosis was the most frequent clinical outcome according to microbiological criteria (*L. monocytogenes* detected in blood) and gastrointestinal symptoms were the most frequent manifestations. Remarkably, only 12% of cases were pregnancy-associated (gestational and neonatal listeriosis) and few cases were severely immunocompromised, despite being primarily risk groups affected by invasive listeriosis. Such clinical paradox may have been influenced by an increased sensitivity of clinical detection partly because of the media attention.

Contrary to estimates from other large listeriosis outbreaks, a relatively low CFR (1.5%) was found in this outbreak. For example, CFR was 6.3% (7/112) in the aforementioned outbreak of invasive listeriosis in Germany. In Denmark, CFR in a nationwide outbreak in 2013–14 was 41.5% (17/41) [16], and a CFR of 13.0% (14/108) was reported in a multistate outbreak in the United States (US) in 1998 [17]. The listeriosis outbreak causing the highest recorded death toll occurred in South Africa from 2017 to 2018 and had a CFR of 26.5% (193/728) [18]. However, several caveats should be considered when comparing such rates. Firstly, this outbreak included cases without symptoms of invasive listeriosis, which are not routinely notified in other epidemiological surveillance systems. Secondly, regarding outcomes, pregnancy losses were classified as sequelae instead of deaths. Thirdly, not all food equally supports *L. monocytogenes* growth, which varies depending on the type of product, its preparation, storage and consumption. Disparities exist even among RTE products, as shown by the values of *L. monocytogenes* CFU/g in food samples observed in this study. This is in contrast with findings from the outbreak in Germany (all unopened blood sausage samples below 100 CFU/g) in which contamination likely occurred after production [14]. Finally, clinical features of infected individuals constitute one of the strongest determinants of outcome severity. Although the three patients who died in this outbreak were above the age of 70 years and one had a malignant neoplasm, most cases reported herein were mostly healthy with a median age of 44 years. This profile does not generally coincide with that of cases from the cited studies. For example, the Denmark outbreak comprised hospitalised patients with a median age of 71 years, more than half of whom had a cancer diagnosis. Nevertheless, despite the relatively low CFR calculated in this study according to the case definitions, the impact of the outbreak was considerable, given that an additional five pregnancy losses were accounted for.

The median incubation period was 1 day. Acknowledging the scarcity of data on the incubation period from large food-borne outbreaks of listeriosis, this finding is to some extent consistent with those reported in such outbreaks [19-21]. Almost all cases were diagnosed via blood cultures and three of four patients presented with gastroenteritis; both bacteraemia and gastroenteritis have been linked to a short incubation period, with a median duration of 1 to 7 days [22,23] and 2 days [23], respectively. Furthermore, after the media spread the public health alert, it is likely that patients were well aware of the outbreak and sought medical care earlier. Other factors that may have contributed to the brief incubation period observed include the high exposure dose, influenced by the degree of contamination by *L. monocytogenes*, the amount of food ingested, the way it was prepared and consumed (e.g. when food establishments sold sandwiches of stuffed pork, people did not always report eating or refrigerating them immediately), and the environmental conditions in summer.

As RTE products can be eaten without previous heating and their consumption is on the rise [24], they are quickly becoming a relevant vehicle for listeriosis outbreaks [25]. In Andalusia, stuffed pork is very popular locally, as it is a low-cost, traditional RTE food. It consists of a cold cut of roasted pork with garlic, spices and salt. Often consumed in summer and on special occasions, it constitutes one of the most popular ‘tapas’ and is a common ingredient in aperitives and sandwiches. The brands of stuffed pork from Facility X were also among the most popular brands in at least three of the provinces affected by this outbreak (Seville, Huelva and Cadiz); however, products from these brands were rarely sold in the rest of Spain.

Beyond risks attributable to RTE products, optimal food safety measures to avert *L. monocytogenes* outbreaks have not been fully elucidated. While some countries have implemented more strict regulations (e.g. the US follows a zero-tolerance policy [26]), prevention of food safety incidents requires the convergence of multiple elements, such as critical control points hazard analysis or appropriate employee training in food processing plants, as most food-borne illnesses stem from poor worker hygiene practices [27]. In this outbreak, however, there was no evidence of such practices concerning workers, but poor hygiene conditions in Facility X could have explained the contamination of meat products, which might have happened after thermal treatment and before their delivery to grocers or sellers. Furthermore, there is a need for improvement with respect to food distribution records. Although the collaboration between food security professionals and public health professionals in this outbreak was adequate, the identification of all the establishments to which the stuffed pork had been distributed was not as fast as desired. In the study region, this public health alert boosted the development of a three-step plan for *L. monocytogenes* control [28].

Our findings illustrate the benefits of combining epidemiological data and WGS to identify and control food-borne outbreaks, and underscore the importance of cross-border collaboration — through the EU decision 1082/2013 [29] and the 2015 International Health Regulations— for identifying new cases and sharing results among countries. The extent of international transmission reported by Moura et al. [30] suggests that some outbreaks caused by *L. monocytogenes* go undetected, emphasising the need to improve the coordination between surveillance information systems (microbiology, food safety and human health). To tailor the response to listeriosis, we recommend investigating food-borne transmission in all laboratory-confirmed cases and limiting the exposure window to 10 days before the diagnosis for cases presenting with gastroenteritis.

This study is not free of limitations. Firstly, the actual number of confirmed cases was likely underestimated because of the following reasons: asymptomatic and mild cases do not always seek medical attention, non-specific culture mediums are poorly sensitive to detect *L. monocytogenes*, not every isolate was available for WGS typing and data were missing for 11 cases detected in the six Spanish regions outside of Andalusia. Nevertheless, we believe this underestimation did not have much impact on the investigation since most of the positive test results came from blood samples. Moreover, the implementation of a communication campaign increased awareness about the public health alert. Secondly, we could not access electronic records from patients hospitalised in private hospitals (11.3%), which impairs the quality of the clinical data. Thirdly, we used a complex definition for confirmed cases that included food consumption, while ideally case definitions should be restricted to disease outcome categories and demographic characteristics. Fourthly, we did not show genetic relatedness analyses as genome comparisons according to Ruppitsch’s core genome MLST scheme are being assessed in a different work in the context of the LISMOAN project. Fifthly, a specific food vehicle was suspected by identifying a common exposure to a product across epidemiological interviews, owing to the high proportion of confirmed cases interviewed (95.2%). However, recall bias cannot be ruled out and food exposure was assessed at a single time point, as there were no follow-up interviews. Finally, during outbreak investigations, case–control comparisons may allow the identification of food exposures, but they are resource-intensive. In this outbreak, the exponential epidemic growth and the high number of hospitalisations prevented local epidemiologists from conducting a proper analytical study.

## Conclusion

This outbreak with 207 confirmed cases and three fatalities was among the largest reported in Europe. Stuffed pork from a single facility contaminated with *L. monocytogenes* ST388 was identified as the source of infection by epidemiological and trace-back investigations. Notification of new cases declined abruptly after control measures, including a large food recall, were applied. Efforts to incorporate WGS in outbreak investigations and coordinate different sectors at regional and national levels are essential for the prevention and control of listeriosis.

## Supplementary Data

480000 Supplement
