# Erratum for Euro Surveill. 2022;27(43)

**DOI:** 10.2807/1560-7917.ES.2022.27.44.221103e1

**Published:** 2022-11-03

**Authors:** Eurosurveillance editorial team

**Affiliations:** 1 European Centre for Disease Prevention and Control (ECDC), Stockholm, Sweden

A correction was made to the article ‘Listeriosis outbreak caused by contaminated stuffed pork, Andalusia, Spain, July to October 2019’ by Fernández-Martínez et al. published on 27 October 2022. The level of significance was noted as ‘p values  ≥  0.05’ in the originally published version. This was corrected to ‘p values  ≤  0.05’ on 28 October 2022. We apologise for any inconvenience this may have caused.

