# Supplementary material for: Listeriosis outbreak caused by contaminated stuffed pork, Andalusia, Spain, July to October 2019
Source: Euro Surveill. 2022 Oct 27;27(43):2200279. doi: 10.2807/1560-7917.ES.2022.27.43.2200279 (PMC9615414; doi:10.2807/1560-7917.ES.2022.27.43.2200279)

"This supplementary material is hosted by Eurosurveillance as supporting information alongside the article **“Listeriosis outbreak due to contaminated stuffed pork, Spain, 2019”**, on behalf of the authors, who remain responsible for the accuracy and appropriateness of the content. The same standards for ethics, copyright, attributions and permissions as for the article apply. Supplements are not edited by Eurosurveillance and the journal is not responsible for the maintenance of any links or email addresses provided therein."

Supplement S1. Listeriosis epidemiological questionnaire employed by professionals from the Epidemiological Surveillance System of Andalusia (SVEA).

Note: All information provided is confidential. This information will be used to determine the cause of the disease in the interviewee and to prevent further cases in the population.

1. Data concerning the notifier and the notification

Case identifier: ______________________________

Hospital/Primary care centre notifying: ___________________________

Date of the first case notification: _______________

Date of the questionnaire: _______________

Name of the notifier: ____

Name of the interviewee: _______________________________________

2. Data concerning the patient

Patient’s full name: _______________________________________

Date of birth: _______________ Sex: _______________

Age, in years: ___ Age, in months (if <2 years): ___ Age, in days (if <1 month): ___

Place of residency:

Country: ___________________ Autonomous community: _____________________

Province: __________________ Municipality: _______________________________

3. Data concerning the disease

Date of the case: _______________

Date of onset of symptoms: _______________

Place of the case:

Country: ___________________ Autonomous community: _____________________

Province: __________________ Municipality: _______________________________

Imported case: ______________

Symptoms	Yes	No	Unknown
Diarrhoea (three or more loose stools in 24 h)			
Headache			
Vomiting			
Chills			
Nausea			
Muscle aches			
Fever			
No symptoms			
Other symptoms: _________________________________			

3.1. Clinical syndrome

o Fever o Meningitis or meningoencephalitis o Bacteraemia or sepsis

o Local infections (arthritis) o Endocarditis/abscesses

Other: ____

3.2. Clinical syndrome (for pregnancy-associated cases)

o Fever o Influenza-like illness o Abortion o Premature birth

o Stillbirth (after the 22th gestational week and weight ≥500 g)

Other: ____

Gestational week: ______

Pregnancy outcome:

o Still pregnant o Spontaneous abortion o Induced abortion

o Stillbirth (after the 22th gestational week and weight ≥500 g)

Date of birth (new-born): _______________

Non-specific gastroenteritis (within 10 days after the date of onset of symptoms): ____

3.3. Clinical syndrome (for neonatal cases)

o Bacteraemia/sepsis o Granulomatosis infantiseptica o Dyspnoea

o Meningitis or meningoencephalitis o Mucocutaneous lesions

o Stillbirth (after the 22th gestational week and weight ≥500 g)

Other: ____

Hospitalisation: _______ Was it due to listeriosis? _______

Hospital: ____

Date of admission: _______________ Date of discharge: _______________

Death: _______ Was it due to listeriosis? _______

Date of death: _______________

Place in which transmission is suspected: ____________________________________
Country: ___________________ Autonomous community: _____________________

Province: __________________ Municipality: _______________________________

Imported case: ______________ Case classification: ______________

Outbreak association: _________ Outbreak identifier: __________________

Autonomous community in which the outbreak was declared: _____________________

4. Laboratory data

Date of sample reception: _______________

Date of diagnosis: _______________

Laboratory-confirmed causative agent: o *Listeria monocytogenes*

Serotype: ______

Serogroup (according to polymerase chain reaction [PCR]): ______

Sample (select only those with a positive result to *L. monocytogenes*):

o Conjunctival exudate o Nasopharyngeal exudate o Cerebrospinal fluid o Blood

o Other normally-sterile tissues. Please specify: _______________________________

o Non-sterile tissues. Please specify: ____

Diagnostic method:

o Isolation o PCR o Genome sequencing

Sample sent to the National Reference Laboratory of Reference (NRL): ______________

Notifier identifier (NRL): ______________ Sample identifier (NRL): ______________

5. Data concerning risk factors

High-risk occupation:

o Veterinary o Food industry worker o Farmer o Hotel/restaurant worker

o Obstetrics nurse o Other: ____

Predisposing factors (select all that apply):

o Pregnancy o New-born o Immunodeficiency o Other: __________________

Suspected route of transmission:

o Ingestion of food (excluding water) o Maternofoetal transmission o Healthcare-associated

o Associated with elaboration of foods that support *L. monocytogenes* growth

o Associated with contact with animals for slaughter

Suspected exposure site:

Transport: ___________________________________

Congregate setting: ___________________________________

Soup kitchen: ___________________________________

Other: ___________________________________

Family environment: ___________________________________

Travel information:

History of travel during the incubation period: ___________ Place:

Country: ___________________ Autonomous community: _____________________

Province: __________________ Municipality: _______________________________

Departure date: _______________ Return date: _______________

6. Food questionnaire

A. In the 4 weeks previous to the onset of symptoms (from _____________ to _____________):

Did you stay in a congregate setting (nursing home, mental health institution…)? ___________

Name of the setting: ____

Did you have contact with domestic animals (dogs, cats, fish…) or their products? ___________

Please specify: ____

B. Where did you purchase the food that you consumed within the 4 weeks previous to the onset of symptoms? Please indicate not just the usual places (e.g., large supermarkets chains) but every store where you may have acquired food products.

Name of the store	Address

C. In the 4 weeks previous to the onset of symptoms, did you eat out (e.g., to have lunch, to have 'tapas', to celebrate a special occasion)?

Name of the establishment	Address

D. Dietary habits

Are you a vegan or vegetarian? ___________

Are you allergic to any food? ___________ Please specify: ________________________

Have you been on a special diet (e.g., Halal, Kosher…) within the 4 weeks previous to the onset of symptoms? ___________ Please specify: ____

Have you eaten any home-made food (e.g., from your kitchen garden)? ___________

Please specify: ____

Have you eaten non-ready-to-eat foods without cooking them (e.g., deli meats)? ___________

Please specify: ____

E. Have you eaten the following foods within the 4 weeks previous to the onset of symptoms? For each food, please answer whether you consumed it, whether you probably consumed it, or whether you did not consume it. Consumption encompasses eating a food product alone or as a part of any dish, including salads, sandwiches, ‘tapas’ and mixed platters.

Note: if the interviewee is not sure about having consumed a particular food, DK (does not know) must be selected.

Food	Consumption				Details	Place of acquisition
	Yes	No	Probably	DK		
MEAT PRODUCTS						
**Deli turkey**						
Packaged						
Not packaged						
**Deli chicken**						
Packaged						
Not packaged						
**Deli pork** (including boiled ham, bologna, ‘Spam’…)						
Packaged						
Not packaged						
**Boar head**						
Packaged						
Not packaged						
**Fresh (Spanish) chorizo**						
Packaged						
Not packaged						
**Other cured deli meats** (including serrano ham, salami, ‘cecina’, [Spanish] chorizo…)						
Packaged						
Not packaged						
**Pâté (not preserved)** (excluding goose/duck pâté)						
Packaged						
Not packaged						
**Goose/duck pâté, foie-gras, mousse, mi-cuit (not preserved)**						
Packaged						
Not packaged						
**Frankfurters**						
**Packaged sandwiches**						
**Pre-cooked meat products**						
**Other meat products**						
DAIRY PRODUCTS						
**Brie**						
**Camembert**						
**Blue cheese** (e.g., Roquefort)						

**Mozzarella**						
**Latin-style cheese**						
**Burgos cheese**						
**Soft or semi-soft cheese**						
**Raw milk cheese**						
**Other types of cheese**						
**Butter**						
**Raw milk**						
**Pasteurised/ UHT milk**						
**Ice cream**						
**Yoghurt**						
**Curd**						
**Other dairy products** (e.g., cottage cheese)						
FISH AND SEAFOOD						
**Prawns/ shrimps***						
**Shellfish*** (e.g., mussels, oysters, clams)						
**Surimi, 'gulas', fish sticks***						
**Smoked/cured fish** (e.g., salmon, eel, mackerel)						
**Raw fish** (e.g., ceviche, sushi, sashimi)						
**Other fish or seafood**						
VEGETABLE PRODUCTS						
**Sprouts (not preserved)** (e.g., soybean, alfalfa)						
**Lettuce/ mesclun**						
**Refrigerated ready-to-eat salads**						
**Raw mushrooms**						
**Aromatic herbs** (e.g., coriander, basil, parsley)						
**Other vegetable products**						
FRUITS						
**Sliced fruit** (e.g., melon, watermelon)						
**Unpasteurised juice**						
**Other types of fruit**						

PRE-COOKED DISHES AND OTHER FOODS						
**Pre-cooked dishes, heated before consumption**						
**Pre-cooked dishes, ready-to-eat**						
**Ready-to-eat foods** (i.e., not requiring further cooking or processing)						
**Foods acquired in food markets**						
**Foods acquired in take-away establishments**						
**Street food**						

*Excluding those heated before consumption.

7. Other relevant data

Do you suspect that your symptoms are related to any food in particular? ___________

Please specify: ____

Do you know any person with similar symptoms associated with consumption of any food in particular? ___________

Do you know any person who experienced diarrhoea within the last month? ___________

Do you usually eat any type of food that does not require much cooking, such as frozen vegetables, meat, raw milk, packaged salads? ___________

Please specify: ____

Do you usually cook or heat raw foods, such as vegetables, long enough? ___________

Please specify which foods: ____

May our Food Safety colleagues go to your home to take samples of suspected food items? ___________

Have you assisted to any group event (such as weddings, baptisms, family or work meetings…) in the last two months? ___________ Please specify: ______________________________

Do you know any person who assisted to said event and developed gastroenteritis?

If you have any doubts or questions, or if you remember any relevant information, you can contact us on the following number: ___________________________________

ADDITIONAL COMMENTS:

8. Foods items sampled

Type of food confirmation (i.e., type of evidence by which the suspected item has been deemed as the food vehicle):

o Epidemiological o Laboratory o Epidemiological and laboratory evidence

Confirmed causative agent in food (select only in the event of laboratory confirmation):
o *Listeria monocytogenes*

Serotype: ______

Serogroup (according to polymerase chain reaction [PCR]): ______

Diagnostic method:

o Investigation o Quantification (count) o PCR o Genome sequencing

9. Case categorisation

Case categorisation (select only one option):

o Probable o Confirmed

Criteria for case classification:

Clinical: ______

Epidemiological: ______

Laboratory: ______

ADDITIONAL COMMENTS (please include all relevant information not detailed previously):

Stuffed pork and any meat products from facility X were sequentially added to this questionnaire (section 6) since they were first suspected as the food vehicle and the source of infection, respectively. An emphasis was placed on acute gastroenteritis within 10 days of consumption (section 3), as it was the most frequent clinical presentation in this outbreak. Epidemiological case definition criteria were modified to include consumption of stuffed pork or any meat products from facility X, and the outbreak time window (section 9).

This questionnaire has been translated to English. The original version is available at: https://www.juntadeandalucia.es/export/drupaljda/Encuesta_12042021_PB_Encuesta%20LISTERIOSIS-1.pdf

Supplement S2. Multivariable logistic regression model: factors associated with a shorter incubation period of listeriosis (defined as 2 days or fewer).

Risk factor	Crude OR (95% CI)	Adjusted OR (95% CI)	p-value
Gastrointestinal presentation	6.70 (3.14-14.76)	6.73 (3.00-15.65)	<0.001*[(1)]
Days elapsed since the outbreak start	1.09 (1.05-1.14)	1.09 (1.05-1.14)	<0.001*[(1)]
Immunosuppression	0.64 (0.22-1.96)	0.56 (0.17-1.93)	0.338[(1)]
Age, years	1.01 (0.99-1.02)	1.01 (0.99-1.03)	0.353[(1)]
Male sex	1.28 (0.67-2.51)	1.19 (0.55-2.62)	0.660[(1)]

CI: confidence interval. OR: odds ratio. *p-value <0.05 of Wald's test (1).

Supplement S3. Epidemiological and clinical characteristics of historically-associated cases of listeriosis.

Variable	Historically-associated confirmed cases (n = 16) N (%), x (s)		
	Total (n = 16)	Women (n = 7, 43.7%)	Men (n = 9, 56.3%)
Age, years: x (s)	51.4 (20.3)	50 (14.1)	52.4 (24.8)
Age categories, years			
0-4	1 (6.3%)	0 (0%)	1 (11.1%)
5-19	0 (0%)	0 (0%)	0 (0%)
20-39	3 (18.9%)	2 (28.6%)	1 (11.1%)
40-59	7 (43.7%)	3 (42.9%)	4 (44.4%)
60-79	4 (25%)	2 (28.6%)	2 (22.2%)
80-100	1 (6.3%)	0 (0%)	1 (11.1%)
Province			
Seville	13 (81.3%)	6 (85.7%)	7 (77.8%)
Cadiz	2 (12.5%)	0 (0%)	2 (22.2%)
Huelva	1 (6.3%)	1 (14.3%)	0 (0%)
Food exposure			
Stuffed pork	1 (6.3%)	1 (14.3%)	0 (0%)
Unknown	15 (93.7%)	6 (85.7%)	9 (100%)
Immunosuppression	4 (25.0%)	0 (0%)	4 (44.4%)
Infection site			
Blood	10 (62.5%)	4 (57.1%)	6 (66.7%)
Cerebrospinal fluid	4 (15.0%)	3 (42.9%)	1 (11.1%)
Unknown	2 (12.5%)	0 (0%)	2 (22.2%)
Hospitalisation	15 (93.8%)	7 (100%)	8 (88.9%)
Outcome			
Cure	3 (18.8%)	3 (42.9%)	5 (55.5%)
Sequelae	1 (6.3%)	1 (14.3%)	0 (0%)
Death	1 (6.3%)	0 (0%)	1 (11.1%)
Unknown	11 (68.9%)	3 (42.9%)	3 (33.3%)

Missing values are presented as "unknown". Data are presented as mean (x) and standard deviation (s) for quantitative variables and absolute frequency (n) and relative frequency (%) for qualitative variables. Illness onset dates ranged from 27 November, 2018 to 7 June, 2019, with the maximum number of historically-associated cases observed in February 2019.

Supplement S4. Incidence per 100,000 population, by health district.

Province	District	Cases	Population	Incidence
Huelva	Condado-Campiña	24	153,728	15.6
Seville	Aljarafe	41	386,444	10.6
Seville	South Seville	41	425,355	9.6
Seville	North Seville	26	276,675	9.4
Seville	Seville	52	688,711	7.6
Cadiz	Jerez-Costa Noroeste	8	340,288	2.4
Seville	East Seville	3	170,158	1.8
Huelva	Huelva-Costa	5	287,273	1.7
Cadiz	Campo de Gibraltar	2	269,294	0.7
Malaga	Axarquía	1	166,355	0.6
Malaga	Costa del Sol	2	534,622	0.4
Cadiz	Bahía de Cádiz-La Janda	1	513,114	0.2
Malaga	Malaga	1	623,276	0.2

Supplement S5. CFU/g of *L. monocytogenes* in contaminated food samples.

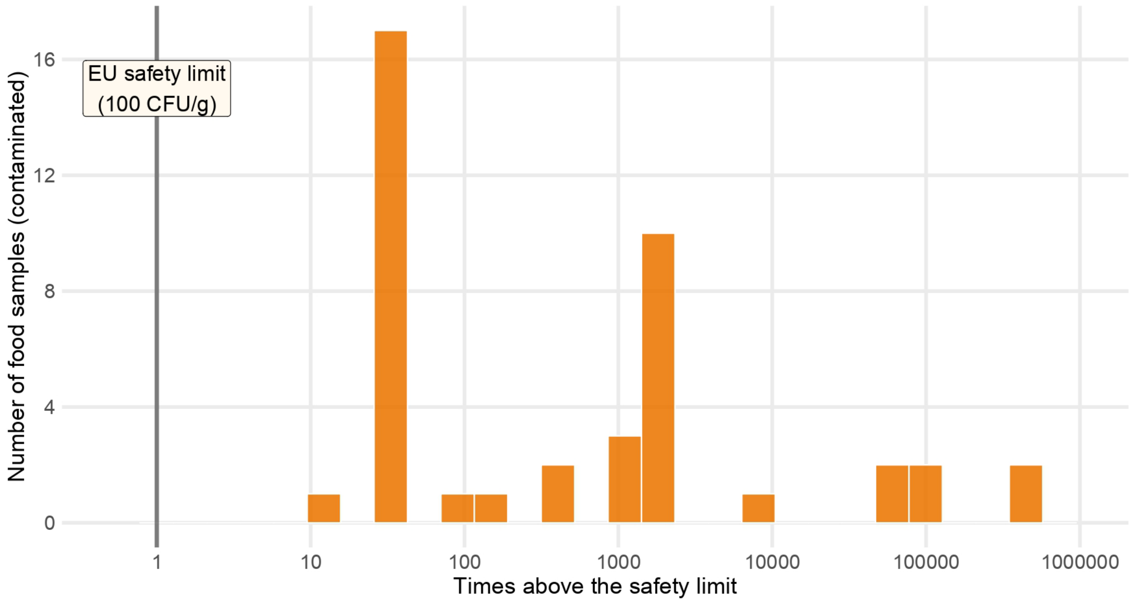

Supplement: Supplement [file 22-00279_RUIZ-MONTERO_SUPPLEMENT.pdf]
